# COVID-19 Predictors of Morbidity and Mortality

**DOI:** 10.7759/cureus.59017

**Published:** 2024-04-25

**Authors:** Gurdeep Singh, Caroline M Tuczinski, Reshma S Thatipelly, Habib Aminy, Numair Tahir

**Affiliations:** 1 Endocrinology, Diabetes, and Metabolism, Our Lady of Lourdes Memorial Hospital, Binghamton, USA; 2 Endocrinology, Harpur College of Arts and Sciences, Binghamton University, Binghamton, USA; 3 Family Medicine, Our Lady of Lourdes Memorial Hospital, Binghamton, USA

**Keywords:** covid-19 mortality, risk factors of covid-19, comorbidities and covid-19, morbidity and mortality, covid-19

## Abstract

Background and objectives

There are multiple factors and comorbid conditions that can impact the outcomes of COVID-19. This study aimed to assess how patients with certain comorbidities and risk factors were affected by COVID-19.

Methodology

This retrospective study involved 578 inpatients who presented to the emergency room (ER) due to COVID-19 infection, diagnosed with COVID-19 between 2020 and 2021. This research takes note of COVID-19 severity, particularly in individuals with comorbidities such as chronic obstructive pulmonary disease (COPD), diabetes mellitus (DM), chronic kidney disease (CKD), coronary artery disease (CAD), and hypertension.

Results

A two-sample t-test found that age was a significant factor affecting hospital length of stay (LOS) and mortality. An ANOVA analysis of race, DM, and CAD showed a significant effect on LOS (p-values = 0.005, 0.01, and 0.01, respectively) but not on mortality and intubation. White patients had an increased LOS compared to other ethnicities. CKD and hypertension significantly affect mortality and LOS. However, COPD did significantly influence all three variables: mortality, intubation, and LOS (p-values = 0.005, 0.013, and 0.01, respectively). A multiple ANOVA test showed that COPD, hypertension, and CKD had a significant effect on mortality, intubation, and LOS (p-values = 0.014, 0.004, and 0.01, respectively). DM showed weaker significance on mortality, intubation, and LOS (p-value = 0.108).

Conclusions

Patients with all three comorbid conditions (COPD, hypertension, and CKD) had increased length of stay, intubation, and mortality; thus, appropriate measures including close observation and early intervention may be necessary to prevent mortality in these patients.

## Introduction

The COVID-19 pandemic started in 2020 with an outbreak in Wuhan, China, and has since claimed 6,893,190 deaths [1,2]. The virus can spread through droplets, airborne particles, and personal contact with others [3]. Common presentations of COVID-19 include, but are not limited to, fever, chills, body aches, anosmia, dysgeusia, sore throat, cough, and shortness of breath [4]. Manifestations of COVID-19 range from asymptomatic to critical, requiring intubation and intensive care unit admission [5].

There are multiple factors and comorbid conditions that can impact the outcomes of COVID-19. Several studies have shown how chronic illnesses such as chronic obstructive pulmonary disease (COPD), diabetes mellitus (DM), chronic kidney disease (CKD), coronary artery disease (CAD), and hypertension (HTN) increased COVID-19 severity [6-10]. Certain risk factors such as old age, high smoking frequency, and obesity are also associated with COVID-19 severity and mortality rate [10-17]. Other studies have noted gender differences between COVID-19 patients, finding that men with COVID-19 are at higher risk for worse outcomes and death, independent of age [13,18,19]. Lastly, previous studies showed that the African American and Asian races were at a higher risk for developing severe COVID-19 symptoms than the White race [20].

This study aimed to assess how patients with certain comorbidities and risk factors were affected by COVID-19. Morbidity is attributed to the comorbidities listed in the methodology section below.

## Materials and methods

This was a retrospective study that involved 578 patients who presented to the ER of Our Lady of Lourdes Memorial Hospital, Binghamton, NY, with COVID-19 between April 21, 2020, to June 24, 2021. The patients needed a documented positive COVID-19 test for inclusion in the study. Patients without documented comorbid conditions and intubation status were excluded from the study.

We collected patient demographics including age, sex, race, ethnicity, weight, height, body mass index (BMI), and smoking status. We consider comorbidities such as COPD, DM, CKD, CAD, and HTN.

The study protocol was approved by Our Lady of Lourdes Memorial Lourdes Hospital IRB (IRB ID 306); informed consent was not obtained due to the retrospective nature of the study.

During the study’s timeframe, laboratory results to confirm initial COVID-19 diagnosis in the hospital were obtained utilizing either ID-NOW technology (Nucleic Acid Amplification Testing), Panther (PCR testing), or BIOFIRE Respiratory Panel.

Inclusion criteria

All patients admitted to the ER, emergency floor, or ICU due to COVID-19 infection and who tested positive were included in the study.

Exclusion criteria

Patients who tested positive for COVID-19 in the outpatient setting and who were admitted to Our Lady of Lourdes Memorial Hospital for conditions unrelated to COVID-19 were excluded from the study.

Outcomes

To investigate the impact of age on mortality rates and LOS, two-sample t-tests across two age categories (≥65 years and <65 years) were used. Three one-way ANOVAs were conducted to examine the effects of various factors (race, DM, CAD, gender, BMI, smoking, CKD, HTN, and COPD) on LOS, mortality rates, and intubation. A multiple ANOVA (MANOVA) was also utilized to explore the combined influence of COPD, HTN, CAD, CKD, DM, and smoking on the following three primary outcome variables: mortality, intubation, and LOS.

## Results

The sample consisted of 578 patients with an age range of 1 year to 101 years. Of the sample, 46% were male and 54% were female. Overall, 80% (N= 461) of the population were white, 13% (N=75) were black, 6% (N= 33) were unknown, and 1% (N= 7) were Asian. Out of 578 patients, 23 (4%) patients were intubated, and 39 (7%)patients needed ICU care. Lastly, 65 (11%) out of 578 patients died (Table 1).

**Table 1:** COVID-19 related comorbidity and mortality The data are presented as N (%).

COVID-19 comorbidities/mortality	Number of patients (%)
Intubation	23 (4%)
ICU admission	39 (7%)
Mortality	65 (11%)

As shown in Table 2, the mean BMI was 31.7 ±9.3 (SD), the mortality rate was 30.8±8.7, and the intubation rate was 36.4±11.9. The mean age affected was 59.1 ±22.5. The mortality rate affected the age mean of 77.2±12.7(p<0.001), and the intubation rate was noticed in the age mean of 62.9 ±15.5 (p=0.22). The rate of mortality was close between the male and female genders at 10.2% (N=32) of females and 12.5% (N=33) of males (p=0.38). Similarly, the rate of intubation was close between males and females at 3.2% (N=10) for females and 4.2% (N=11) for males (p=0.83). Our data predominantly consisted of the white population at 80% (N=461) with a mortality rate of 11.9% (N=55; p=0.18) and an intubation rate of 3.5% (N= 16; p=0.91). For smokers, mortality occurred at a rate of 11.3% (N=16; p=0.90) and intubation at 4.3% (N=6; p=0.93). Other patient-related information was collected, such as smoking, DM, CKD, CAD, HTN, and COPD.

**Table 2:** Impact of demographics on COVID-19 severity The data are presented as mean ± SD, N (%), and p-values.

		Overall	Mortality			Intubation			LOS
			Yes	No	p	Yes	No	p	p
Age, mean (SD)		59.1 (22.5)	77.2 (12.7)	56.8 (22.5)	<0.001	62.9 (15.5)	58.7 (23.1)	0.22	<0.001
BMI, mean (SD)		31.7 (9.3)	30.8 (8.7)	31.8 (9.4)	0.38	36.4 (11.9)	31.48 (9.17)	0.06	0.07
Gender, N (%)	Female	315 (54.4)	32 (10.2)	282 (89.5)	0.38	10 (3.2)	304 (96.5)	0.83	0.73
	Male	264 (45.6)	33 (12.5)	229 (86.7)		11 (4.2)	251 (95.1)		
Ethnicity, N (%)	White	461 (80.0)	55 (11.9)	404 (87.6)	0.18	16 (3.5)	443 (96.1)	0.91	0.005
	Black	75 (13.0)	4 (5.3)	70 (93.3)		3 (4.0)	71 (94.7)		
	Asian	7 (1.2)	2 (28.6)	5 (71.4)		0 (0.0)	7 (100)		
	Unknown	33 (5.7%)	3 (9.1)	30 (90.9)		2 (6.1)	31 (93.9)		
Smoking status, N (%)	Yes	141 (24.6)	16 (11.3)	124 (87.9)	0.90	6 (4.3)	134 (95.0)	0.93	0.97
	No	432 (75.4)	37 (8.6)	364 (84.3)		8 (18.5)	393 (91.0)		

**Figure 1:**
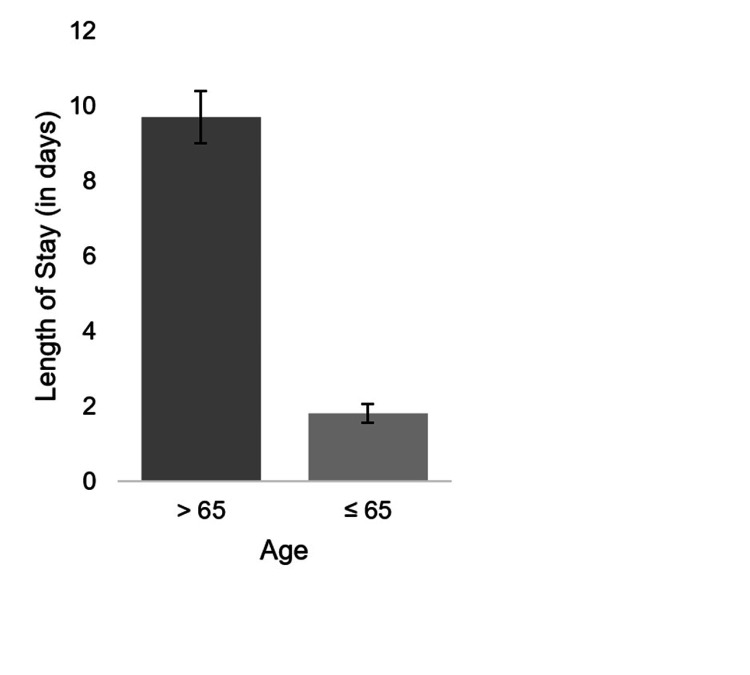
Correlation between age and length of stay for COVID-19 patients Length of stay for patients aged >65 years (M = 9.7, SD = 11.2, N = 261) and ≤65 years (M = 1.8, SD = 4.37, N = 317; p < 0.001). Error bars represent ± 1 standard error. The p-value is considered significant at p < 0.05. The data are presented as N and as mean ±SD.

Using a two-sample t-test, our results found that age is a significant factor in affecting mortality and inpatient length of stay with p < 0.001. For patients ≥65 years (N=261), the mean length of stay (LOS) was 9.7±11.2 days (mean ±SD). For patients <65 years (N =317), LOS was 1.8 ±4.37 days (Figure 1). The second phase of analysis was based on conducting an ANOVA. A statistical technique was used to check if the means of two or more groups are significantly different from each other. Null hypothesis states that population means are equal with a significance level of 95%.

Ethnicity, DM, and CAD were shown to be have a significant effect on LOS (p-values (0.005, 0.01, and <0.001, respectively); however, no significant effect was shown on mortality and intubation (Tables 2, 3). As shown in Table 2, for race, white patients ranked the highest in the mean value of LOS (N=461 patients, 80%; mean=6 days), followed by Asian (N=7 patients, 1.2%; mean=2.71 days) and lastly Black (N=75 patients, 13%; mean=2.2 days). Due to an unbalanced sample, the pooled standard deviation was used to calculate intervals, and the weighted average given to the larger sample size was used. Gender, BMI, and smoking did not show a significant effect on LOS, mortality, and intubation.

**Table 3:** Impact of comorbidities on COVID-19 severity The data are presented as N (%) and p-values. CKD, chronic kidney disease; CAD, coronary artery disease; COPD, chronic obstructive pulmonary disease

Comorbidity	Overall	Mortality		Intubation		LOS	
	N (%)	N (%)	p	N (%)	p	Mean (days)	p
Diabetes	157 (27.2)	22 (14.1)	0.21	10 (6.4)	0.08	7.24	0.01
CKD	66 (11.4)	16 (24.2)	<0.001	2 (3.0)	0.68	13.27	<0.001
CAD	90 (15.6)	15 (16.7)	0.09	4 (4.4)	0.83	9.64	<0.001
Hypertension	277 (47.9)	41 (14.8)	0.01	13 (4.7)	0.40	7.96	<0.001
COPD	46 (15.6)	11 (23.9)	0.005	5 (10.9)	0.01	10.63	<0.001

CKD and HTN are significant factors affecting both LOS and mortality but not intubation (Table 3). Similarly, in Table 3, COPD is a significant factor affecting mortality, intubation, and LOS (p = 0.005, 0.013, and <0.001, respectively).

MANOVA was conducted to study multiple factors (COPD, HTN, CAD, CKD, DM, and smoking) affecting the three dependent variables/outcomes (mortality, intubation, and LOS). MANOVA evaluates if combinations of multiple factors simultaneously affect the dependent variable by a statistically significant level.

COPD, HTN, and CKD were shown to be significant factors affecting the three outcomes (mortality, intubation, and LOS) (p = 0.014, 0.004, and <0.001, respectively). DM showed to be a marginally significant factor affecting the dependent variables, with p = 0.108 (Table 3).

**Figure 2:**
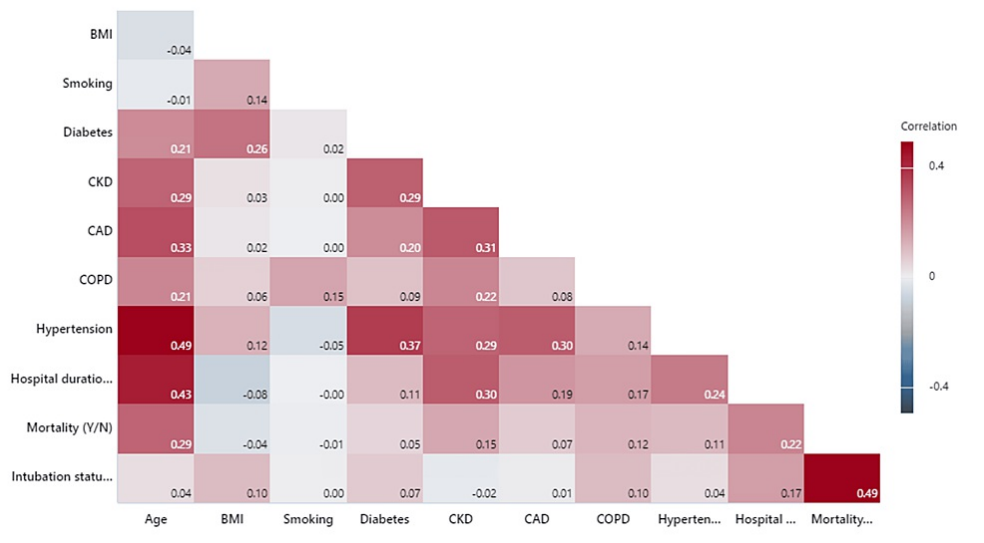
Correlogram The correlogram compares the Pearson correlation coefficients between each pair of variables. Hypertension and age, and intubation and mortality show a strong positive correlation (correlation coefficient=0.49). The p-value is considered significant at p < 0.05. The data are represented as p-values.

Correlogram was created to provide more insight into the data and correlation between several factors (Figure 2). The correlogram statistically compares the Pearson correlation coefficients between each pair of variables. It showed that HTN and age, and intubation and mortality had a strong positive correlation (correlation coefficient= 0.49). Gender was not a significant factor affecting LOS and mortality.

## Discussion

This study primarily focuses on the relationships between comorbidities, risk factors, and race/ethnicity trends that are associated with COVID-19 severity and mortality. Measures of COVID-19 severity were collected by assessing intubation status and hospital length of stay.

Age, gender, smoking, BMI, and race on COVID-19 disease outcomes

Previous research has found a correlation between age and COVID-19 mortality [11-13]. A study by Yanez et al. examined COVID-19 patients in 16 different countries, finding that in patients aged 54 years and younger, mortality was 8.1 times higher than that in patients aged 55 to 64 years and 62 times higher among patients 65 years and older [13]. Research from Kalligeros et al. found the median age of patients diagnosed with COVID-19 to be 60 (52-70) years [11]. Importantly, research from Hagg et al. showed that older age was correlated with in-hospital mortality (hazard ratio [HR] 1.05 per year, 95% confidence interval [CI] 1.01‒1.08) [12].

Our findings show that age increases the risk of intubation, LOS, and mortality, which is also supported by previous research studies [11,13].

Two studies have also found a correlation between patient gender and COVID-19 disease severity and morbidity. Jin et al. concluded that the number of male deaths as a result of COVID-19 was 2.4 times more than that of women (70.3 vs. 29.7%, p = 0.016) [18]. Additionally, the study found that a higher percentage of males were in the deceased group than in the survived group (p = 0.015). A similar study by Qiu et al. also found that 66.6% of those who have died from COVID-19 were male, with a median age of 69.9 years [6].

Smoking is another risk factor in the progression of the novel coronavirus, with research finding that current smokers have a higher mortality rate than non-smokers [10]. Another study by Reddy et al. found that patients with a smoking history were more at risk for higher mortality and need for mechanical ventilation [14], though this study found smoking to have a weak correlation with the variables of intubation, LOS, and mortality (p-values = 0.01, 0.01, and 0.01, respectively). Interestingly, an article from Rossato claims that there is a low prevalence of smokers in COVID-19 patients, with no significant correlation between smoking and disease severity in COVID-19 patients [15].

Furthermore, in our study, BMI measures were found to have a weaker correlation with mortality and hospital duration. However, a systematic review and meta-analysis by Huang et al. showed a notable relationship between higher BMI and risk for hospitalization, ICU admission, invasive mechanical ventilation (IMV) requirement, and death [16]. The findings of two other studies, Gao et al. and Kalligeros et al., support the relationship between obesity and the severity of COVID-19 symptoms. More specifically, the study by Kalligeros et al. revealed that 56.8% of patients with obesity required ICU care and that 47.5% of patients hospitalized were obese [11]. According to the study by Gao et al., obesity is associated with a longer hospital stay (p=0.037) and a greater severity of COVID-19 symptoms (p=0.007) [17].

Race and ethnicity are important factors when it comes to COVID-19 disease outcomes. A study by Sze et al. examined patients from 50 different studies in the United States and the United Kingdom. This research found that patients with Black and Asian ethnicities were at a higher risk of COVID-19 infection than White patients. Patients with Asian ethnicity were also shown to be at a higher risk of intensive therapy unit admission and death [20].

Gender was not a significant factor affecting LOS, intubation, and mortality, which contradicted findings by Jin et al. and Qiu et al. [6,18]. Possibilities for the differences in findings could be due to the type and setting of collecting data. Jin et al. collected a data set from Beijing, China, and Qiu conducted a meta-analysis for analyzing gender differences. However, another study conducted by Danielsen et al. analyzed the gender disparities in the United States, and this study noticed that sex differences did not correlate with mortality risks [19].

BMI did not show any significant effect on LOS, mortality, and intubation. Our results therefore contradict the study conducted by Huang et al., which shows that a higher BMI is associated with risk for hospitalization, ICU admission, IMV requirement, and death [16]. Our results could have been due to disproportionate obesity rates among the population sample.

Another factor that did not influence LOS, mortality, and intubation was smoking, which directly contradicts the studies by Alqahtani et al. and Reddy et al. [10,14]. Interestingly, a study by Rossato supports our findings that smoking was not impactful in COVID-19 severity. Furthermore, although we found that white patients had a significantly higher LOS, this could be due to our small sample size and lack of diversity in our sample set. Therefore, our results do not match with the study conducted by Sze et al. [20].

Comorbidities and COVID-19 disease outcomes

Multiple studies evaluated the influence of these comorbidities on patient outcomes. A systematic review and meta-analysis by Nandy et al. reported that certain comorbidities had a significant impact on COVID-19 patients as measured by an odds ratio (OR) of serious events such as ICU admission, mechanical intubation, or mortality. The study found the following OR measures in COVID-19 patients: HTN with 2.95, DM with 3.07, COPD with 6.66, and CKD with 5.32 [7].

Another study by Wang et al. found that patients with comorbidities such as HTN, DM, and cardiovascular disease were more at risk for ICU care [9]. A study by Qiu et al. found that the rates of HTN, DM, and cardiovascular disease among deceased coronavirus patients were 38.56%, 22.2%, and 17.54%, respectively [6]. This study shows that HTN, DM, and cardiovascular disease in particular are associated with the risk of mortality in COVID-19 patients.

In our study, DM and CAD had a significant impact on LOS (p-values 0.01 and 0.01, respectively) but not on mortality and intubation, while Wang et al., Nandy et al., and Qiu et al. found that DM impacted mortality and intubation [6,7,9].

Furthermore, HTN increases the risk of intubation, LOS, and mortality. The significant factors affecting mortality were COPD, HTN, and CKD (p-values = 0.014, 0.004, and 0.01, respectively). This finding supports all three studies once again, specifically pertaining to intubation and mortality.

Lastly, we performed MANOVA and found that COPD, HTN, and CKD were significant factors affecting all three outcomes (mortality, intubation, and LOS); thus, patients with all three comorbid conditions should be observed closely as timely intervention can decrease the risk of intubation and mortality.

Limitations

This retrospective study has limitations, including its retrospective nature, use of data from 2020-2021, exclusion of novel COVID-19 strains. Lastly, an unbalanced ethnicity sample size was used which made the white race seem to correlate with longer length of stay. Lastly, an unbalanced ethnicity sample size was used, which made the white race seem to correlate with longer length of stay.

## Conclusions

In our study, we found that age, HTN, DM, COPD, CAD, and CKD can affect intubation, LOS, or mortality related to COVID-19. BMI, gender, and smoking had no significant effect on LOS, mortality, or intubation. DM, ethnicity, and CAD were shown to be significant on LOS but not on mortality and intubation.

MANOVA showed that the presence of all three comorbid conditions (COPD, HTN, and CKD) significantly affected length of stay, intubation, and mortality; thus, appropriate measures including close observation and early intervention may be necessary to prevent mortality in these patients.
